# Flexible Piezoelectric Tactile Sensor Array for Dynamic Three-Axis Force Measurement

**DOI:** 10.3390/s16060819

**Published:** 2016-06-03

**Authors:** Ping Yu, Weiting Liu, Chunxin Gu, Xiaoying Cheng, Xin Fu

**Affiliations:** The State Key Laboratory of Fluid Power and Mechatronic Systems, Department of Mechanical Engineering, Zhejiang University, Hangzhou 310027, China; E-Mails: yuping55@zju.edu.cn (P.Y.); cxgu@zju.edu.cn (C.G.); L_michael@zju.edu.cn (X.C.); xfu@zju.edu.cn (X.F.)

**Keywords:** piezoelectric tactile sensor, three–axis dynamic force, PVDF, flexible, truncated pyramid bump

## Abstract

A new flexible piezoelectric tactile sensor array based on polyvinylidene fluoride (PVDF) film is proposed for measuring three-axis dynamic contact force distribution. The array consists of six tactile units arranged as a 3 × 2 matrix with spacing 8 mm between neighbor units. In each unit, a PVDF film is sandwiched between four square-shaped upper electrodes and one square-shaped lower electrode, forming four piezoelectric capacitors. A truncated pyramid bump is located above the four piezoelectric capacitors to improve force transmission. A three-axis contact force transmitted from the top of the bump will lead to the four piezoelectric capacitors underneath undergoing different charge changes, from which the normal and shear components of the force can be calculated. A series of dynamic tests have been carried out by exerting sinusoidal forces with amplitudes ranging from 0 to 0.5 N in the x-axis, 0 to 0.5 N in the y-axis, and 0 to 1.5 N in the z-axis, separately. The tactile units show good sensitivities with 14.93, 14.92, and 6.62 pC/N in the x-, y-, and z-axes, respectively. They can work with good linearity, relatively low coupling effect, high repeatability, and acceptable frequency response in the range of 5–400 Hz to both normal and shear load. In addition, dynamic three-axis force measurement has been conducted for all of the tactile units. The average errors between the applied and calculated forces are 10.68% ± 6.84%. Furthermore, the sensor array can be easily integrated onto a curved surface, such as robotic and prosthetic hands, due to its excellent flexibility.

## 1. Introduction

Tactile sensing plays an important role in human dexterous manipulation and object perception. This sensing ability depends on two kinds of tactile corpuscles: fast-adapting and slowly-adapting corpuscles in human skin. The fast-adapting corpuscle exhibits sensitivity to dynamic skin deformation of relatively high frequency, and can respond to dynamic events such as making and breaking contact, slippage, and texture perception, while the slowly-adapting corpuscle exhibits sensitivity to lower-frequency skin deformation and can be utilized to detect sustained contact forces [1]. To emulate human perception for sophisticated manipulation, different kinds of tactile sensors have been developed for robotic and prosthetic hands [2,3]. These sensors can be classified according to the sensing mechanism employed, including piezoresistive [4,5], piezoelectric [6,7,8], capacitive [9,10], and optical [11] sensing, *etc*. Among these sensing mechanisms, piezoelectric sensing features excellent sensitivity to dynamic mechanical stimulation over a wide frequency range and, thus, are widely utilized in developing tactile sensors for mimicking the fast-adapting tactile corpuscle [12,13].

For piezoelectric tactile sensors aiming to mimic the fast-adapting tactile corpuscle and applied in limited spaces and curved surfaces, such as robotic and prosthetic hands, the following design requirements should be satisfied: (i) the tactile sensor should be able to measure a dynamic three-axis force in the designed frequency range. In actual cases, when the hand makes contact with an object, forces between the tactile sensor and the object always occur in arbitrary directions, generating normal and shear force components [9]. In addition, slippage and touching perception of surface features are inseparable from tangential motion and accompany with small vibrations [14]. To extract valuable information in these events, tactile sensors should have good dynamic response capability to follow oscillations in both normal and shear force; (ii) sensing elements, microstructures, and package material should be flexible and conform to the three-dimensional surface in order to be deployed on the curved surface of fingertips [9]; and (iii) the fabrication process of the tactile sensor should be compatible with well-developed techniques, such as the surface micromachining process which enables the possibility of miniaturization, good spatial resolution, and mass production [15].

During the last few decades, several piezoelectric tactile sensors have been developed. A piezoelectric tactile sensor array with 8 × 8 sensing units was realized by coupling a polymer polyvinylidene fluoride (PVDF) film to a monolithic silicon integrated circuit. The response of a sensing unit is linear for normal loads spanning 0.008–1.35 N [16]. To improve the scalability and flexibility, a scalable technique based on inkjet printing was provided to pattern electrodes on a piezoelectric film. With this technique, a flexible piezoelectric transducer array sensitive to normal forces was developed [17]. To make a sensor with a better signal-to-noise ratio, faster response, wider bandwidth, and better force sensitivity, a P(VDF-TrFE) (polyvinylidene fluoride-trifluoroethylene) film was directly spin-coated onto the gate area of a metal oxide semiconductor(MOS)transistor [6,7]. The above-mentioned sensor prototypes focused only on dynamic normal force detection. In order to enable shear force sensing, other research groups tried to integrate PVDF sensing elements vertically into an elastomeric structure [18,19]. Furthermore, to simultaneously measure both dynamic normal and shear force components, four piezoelectric sensing elements are symmetrically arranged on the surface of a vertically-placed cylindrical shell. When an external three-axis force is applied at the tip of the shell, the normal force component causes the same electric voltage on all four piezoelectric sensing elements, while the shear force component causes a reversal of voltage on two opposite piezoelectric elements. From signals from the four elements, the direction angles and magnitude of the three-axis force can be calculated [20]. One problem of these shear force and three-axis force sensors is that the piezoelectric sensing elements were vertically placed, thus, the suggested fabrication process could not be compatible with surface micromachining technology and limited further reduction of the sensor size, which is extremely important in space-limited applications, such as robotic and prosthetic hands.

Recently, a structure consisting of a bump layer and a horizontally-arranged sensing array layer has been successfully introduced in capacitive and piezoresistive tactile sensor to measure static three–axis forces [9,21]. Inspired by this, the authors here propose a piezoelectric tactile sensor array meeting the entire three design requirements mentioned above with a designed multilayer flexible structure. Each tactile unit in the sensor array consists of one compliant truncated pyramid bump, which serves as contact force translator; underneath the bump, four separate piezoelectric capacitors based on PVDF are horizontally distributed, which enables three-axis force measurement and makes the fabrication process compatible with the surface micromachining process. Detail design and fabrication of the sensor array are presented, followed by the experimental testing.

## 2. Structure and Operating Principle

### 2.1. Sensor Structure

**Figure 1:**
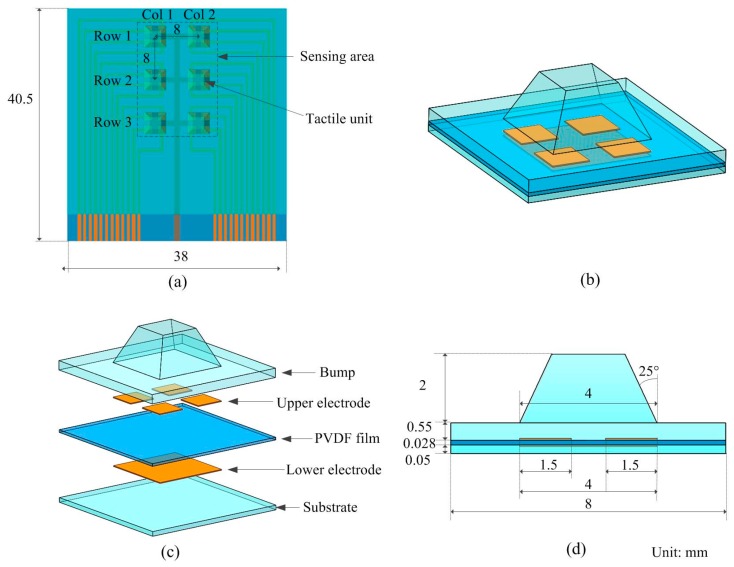
Conceptual diagram of the proposed three–axis tactile sensor array. (**a**) Top view of the array; (**b**) schematic view of a tactile unit; (**c**) exploded view of a tactile unit; and (**d**) cross-sectional view of a tactile unit.

Figure 1 illustrates the conceptual diagram of the proposed three–axis tactile sensor array. Figure 1a is the overview of the sensor array, which contains six three-axis tactile units arranged as a 3 × 2 matrix. The center distance between every two adjacent units is 8 mm. Figure 1b,c illustrate the schematic view and exploded view of a tactile unit. It consists of five layers: a PDMS bump layer, an upper aluminum (Al) electrode layer, a PVDF film layer, a lower Al electrode layer and a PDMS substrate layer. The upper and lower Al electrode layers with thickness of 200 nm are developed through wet etching of commercial two-side metalized PVDF film. The PVDF film is sandwiched between four square-shaped upper electrodes and one square-shaped lower electrode, thus forming four piezoelectric capacitors. Each piezoelectric capacitor has the area of 1.5 × 1.5 mm^2^. A PDMS truncated pyramid bump with dimensions of 4 mm × 4 mm × 2 mm is fixed at the center of the unit with its four bottom corners aligned to the corner of each upper square electrodes, as shown in Figure 1d.

### 2.2. Conceptual Operation Principle

PVDF is a piezoelectric plastic material that generates charges when it is mechanically deformed [22]. The relationship between electric displacement (output charge density), electric field, and mechanical stress is expressed as follows [23]:

(1) $$D_{i}=\sum_{q=1}^{6}d_{iq}T_{q}+\sum_{k=1}^{3}\varepsilon_{ik}^{T}E_{k},i=1,2,3$$

where $D_{i}$ is electric displacement (output charge density) in the i direction, $T_{q}$ is mechanical stress in the q direction, $E_{k}$ is electric field in the k direction, $d_{iq}$ is the piezoelectric constant, and $\varepsilon_{ik}^{T}$ is dielectric constant under constant stress. 

In the event of the PVDF being used in the thickness mode, the relation 1 can be written as follows:

(2) $$D_{3}=d_{33}T_{3}+\varepsilon_{33}^{T}E_{3}$$

In our proposed sensor, the PVDF film mainly works on $d_{33}$ (thickness) mode and with no external electric field. So, the relation 2 can be written as follows:

(3) $$D_{3}=d_{33}T_{3}$$

**Figure 2:**
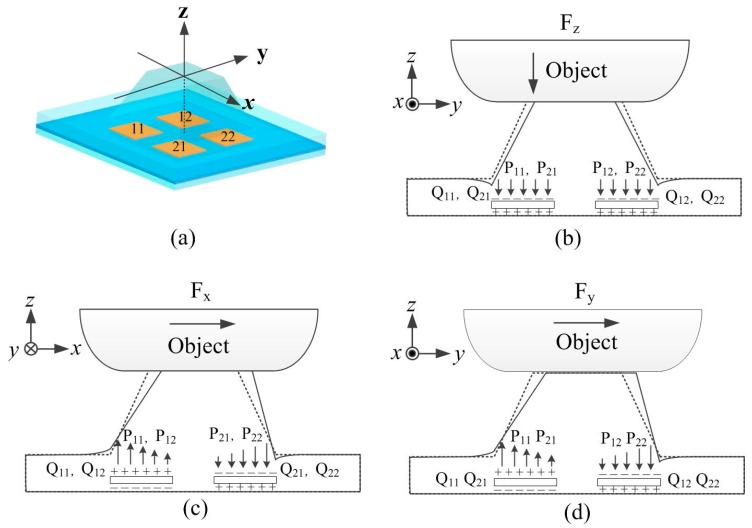
Principle of operation for sensing normal and shear forces in the tactile unit. (**a**) Reference coordinates; (**b**) response to normal force; (**c**) response to shear force in the x-axis direction; and (**d**) response to shear force in the y-axis direction.

Figure 2 shows the principle of operation for sensing a three-axis force in a tactile unit. The applied three-axis force on the bump can be decomposed into one normal force component ($F_{z}$) and two shear force components ($F_{x}\text{ and }F_{y}$) perpendicular to each other, respectively. The normal and shear directions are defined as shown in Figure 2a, in which the normal direction is the through-thickness direction. Under the normal force component, the bump is compressed and the four piezoelectric capacitors are subjected to the same compressive stress ($P_{11},\;P_{12},\;P_{21}\text{ and }P_{22}$). By the piezoelectric effect of the PVDF film, charges ($Q_{11},\;Q_{12},\;Q_{21}\text{ and }Q_{22}$) with the same quantity and polarity (negative) are generated on the upper electrodes of the four piezoelectric capacitors, while equivalent positive charges are generated on the lower electrodes. Under the shear force components, the bump deforms and generates a torque at the fixed end. The two piezoelectric capacitors on the left side are subjected to tensile stress, whereas other two on the right side are subjected to compressive stress, as shown in Figure 2c,d. As a result, the developed charges on the left electrodes and right electrodes are of opposite polarity. Thus, $F_{z}$ can be calculated by taking the average values of $Q_{11},\;Q_{12},\;Q_{21}\text{ and }Q_{22}$, while $F_{x}$ and $F_{y}$ can be calculated from the differences among $Q_{11},\;Q_{12},\;Q_{21}\text{ and }Q_{22}$.

## 3. Fabrication

**Figure 3:**
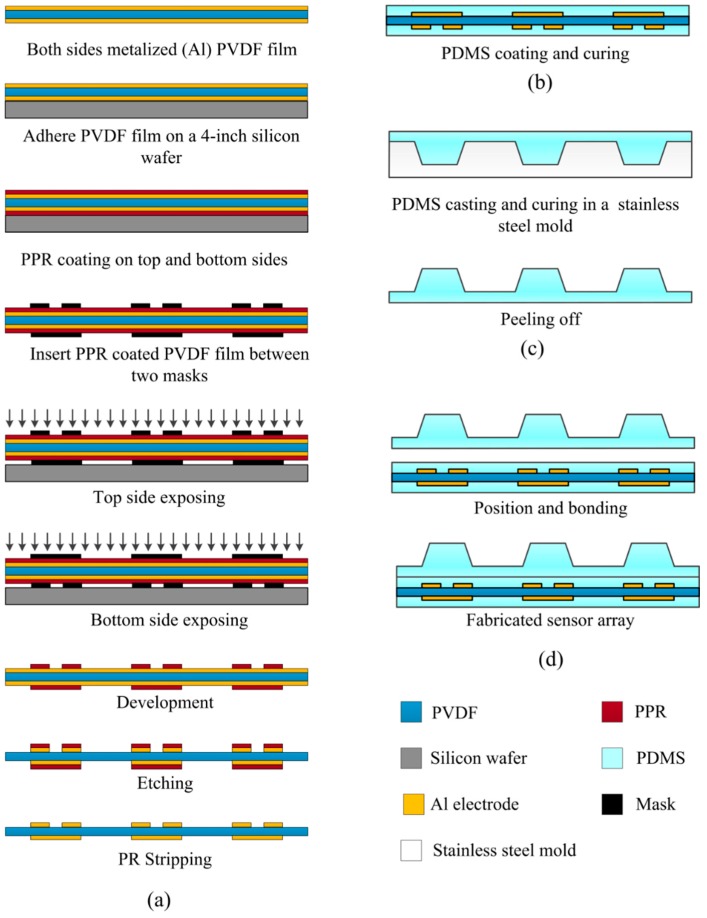
Fabrication process of the proposed three–axis tactile sensor array. (**a**) Electrode layers; (**b**) PDMS protecting layer; (**c**) PDMS bump layer; and (**d**) position and bonding.

The fabrication process of the flexible piezoelectric three-axis tactile sensor array is shown as follows (Figure 3), which is fully compatible with the surface micromachining process.

Step 1: Upper and lower electrode layers patterning, as shown in Figure 3a. A 25 μm-thick, metalized (Cr/Al), and poled PVDF film (Piezotech S.A.S., France) is prepared. The piezoelectricity constant $d_{33}$ of the film is 15 ± 3 pC/N. Firstly, the film is taped on a clean four-inch silicon wafer with care such that the film is stretched uniformly over the wafer. The tape should cover all sides of the PVDF film, overlapping by 2 mm [24]. Secondly, a 2.5 μm-thick RZJ-304 positive photoresist (PPR) is spin-coated onto one side of the PVDF film and then soft baked at 60 °C for 60 min. The reason why soft bake was chosen is that the curie temperature of the PVDF film is only about 135 °C and the polarized electrets are thermodynamically stable up to about 90 °C [25]. For security, soft bake at 60 °C for 60 min is carried out. Thirdly, after cooling down to room temperature, the PVDF film is torn off from the silicon wafer, turned over and then taped again to the silicon wafer as described above. Fourthly, with the same process, PPR is spin-coated onto the other side of the PVDF film. Fifthly, the two-side PPR-coated PVDF film is inserted between two aligned masks which contain patterns for the upper and lower electrode layers. Using an ultraviolet source, PPR on both sides are exposed. Sixthly, the exposed film is developed, rinsed, dried under nitrogen, and baked at 60 °C for 60 min. Seventhly, the PVDF film is immersed into an aluminum etchant, a mixture containing phosphoric acid, acetic acid, nitric acid, and water with weight ratio of 16:4:1:1, for about 10 min to pattern the upper and lower electrodes. At last, the residual PPR is removed by acetone.

Step 2: PDMS film coating, as shown in Figure 3b. The liquid-state PDMS (Sylgard 184, A:B = 10:1 in weight) is spin-coated onto the lower electrode layer at 2000 rpm for 30 s to form a 50 μm-thick PDMS substrate layer and then cured at room temperature for 24 h. The same process is used to form a 50 μm-thick PDMS film on the upper electrode layer for bonding.

Step 3: Bump layer fabrication (Figure 3c). The liquid-state PDMS (10:1) is poured into a stainless steel mold which is manufactured by a milling machine, and then cured at 80 °C for 3 h to replicate the shape of the bump.

Step 4: Sensor array assembling. After being activated by oxygen plasma, the PDMS bump layer fabricated in Step 3, the PVDF layer with patterned electrodes and PDMS coats fabricated in Step 2 are aligned and bonded using a three-axis manual stage (Newport 460 P), as shown in Figure 3d.

**Figure 4:**
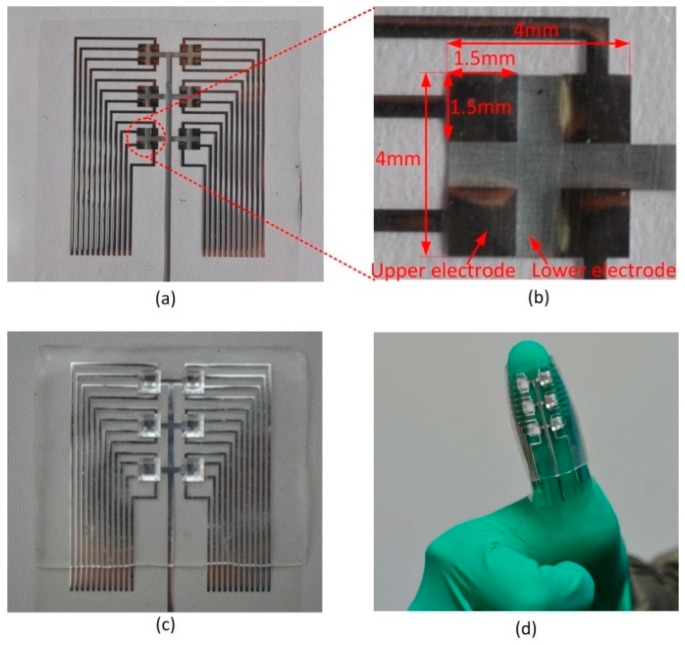
(**a**) Photograph of the fabricated PVDF film with patterned upper and lower electrodes; (**b**) magnified view close to electrodes of a tactile unit; (**c**) photograph of the fabricated three-axis tactile sensor array; and (**d**) photograph of the fabricated sensor array mounted on a curved surface.

Figure 4a shows the fabricated PVDF film with patterned upper and lower electrodes. The electrodes are well fabricated and have high uniformity. Figure 4b is the magnified view close to electrodes of a tactile unit, where four square-shaped upper electrodes aligned well with one square-shaped lower electrode. Figure 4c shows the fabricated piezoelectric three-axis tactile sensor array, which has 6 tactile units with high flexibility, as demonstrated in Figure 4d.

## 4. Measurements and Discussion

### 4.1. Sensor Unit Calibration

**Figure 5:**
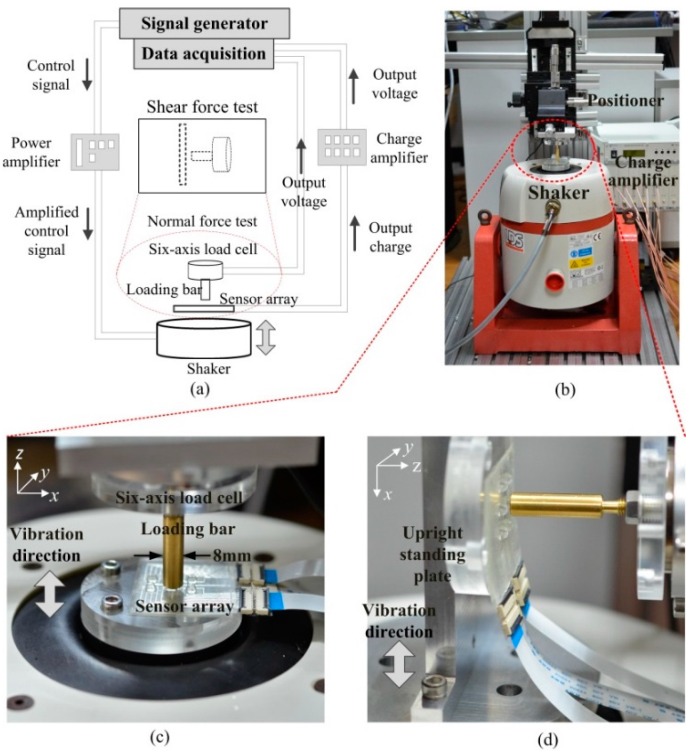
(**a**) Schematic of the experimental setup for sensor unit calibration.; (**b**) photograph of the experimental setup; (**c**) close-up view of the contact between the loading bar and PDMS bump in normal force loading; and (**d**) close-up view of the contact between the loading bar and PDMS bump in shear force loading.

To study the sensing performance of the sensor array, calibration experiments have been conducted. Figure 5a,b show the schematic and photograph of the experimental setup. The tactile sensor array is located on a shaker (LDS V455, Brüel&Kjær, Darmstadt, Germany) which can apply random dynamic forces up to 489 N, with a frequency in the range 5 Hz–7.5 kHz. For normal force loading, the tactile sensor array is horizontally fixed on the shaker’s platform (Figure 5c). As for shear force loading, the sensor array is vertically fixed on the shaker’s platform (Figure 5d), with an upright standing plate. By rotating the sensor array, shear force in any direction can be applied. A round loading bar with a diameter of 8 mm is used to press the bump of a tactile unit against the shaker to add preloading forces. The loading bar is attached to a six-axis load cell (Nano43, ATI Industrial Automation, Apex, NC, USA), which has 0.0078 N resolution, ±36 N measurement range and 0–2800 Hz frequency range. With this strategy, the applied force can be precisely measured by the six–axis load cell. The six-axis load cell is fixed onto a three-axis positioner (M460P, Newport, Miami, FL, USA) to enable the alignment capability between the axis of loading bar and the bump center of a tactile unit. Charges developed by the tactile sensor array are measured by a charge amplifier (DH5862, Donghua Testing Technology Co., LTD, Jingjiang, China) which has 16 channels, 10 mv/pC sensitivity, and 0.3–30 KHz frequency range. Output voltages of both the six-axis load cell and the charge amplifier are collected by a NI-DAQ device (USB-6343, National Instruments Corporation, Austen, TX, USA) and displayed on a computer screen based on Labview software. Parameters of the mechanical stimulus (waveform, frequency, amplitude) are set by a signal generator (33500B, Agilent Technologies, Palo Alto, CA, USA) and then amplified by a power amplifier (PA1000L, Brüel&Kjær, Darmstadt, Germany).

Sinusoidal normal and shear forces with constant frequency (20 Hz) and variable amplitude were applied, respectively, on a tactile unit. Meanwhile outputs from the unit and the six-axis load cell were recorded. Before all the tests, a preload force (about 2 N) should be applied on the top of the tactile unit. Without a preload force, a contact loss between the loading bar and the bump may occur in the negative half cycle of stimulation which leads the applied stimulus to being uncontrolled and generating an erratic force pattern impact on the loading bar surface. It should be noted that piezoelectric transducers only respond to the dynamic forces and, hence, the static preload does not affect the measurements [7]. In addition, all the tests were performed under ambient conditions with a temperature around 20 °C. The experimental procedure is as follows. (1) Normal load test: the tactile array was horizontally located, as in Figure 5c. The shaker was excited to apply a sinusoidal 20 Hz normal force with variable amplitude ($F_{zm}$) in the range of 0–1.5 N on the tactile unit; (2) Shear load test (x-axis): the tactile sensor array was vertically placed, as shown in Figure 5d. The shaker was excited to apply sinusoidal 20 Hz shear forces in the x-axis with variable amplitude ($F_{xm}$) in the range of 0–0.5 N on the tactile unit; (3) Shear load test (y-axis): the tactile sensor array was rotated by 90 degrees along the z-axis. The shaker was excited to apply a sinusoidal 20 Hz shear forces in y-axis with variable amplitude ($F_{ym}$) in the range of 0–0.5 N on the tactile unit. Each test was repeated three times to evaluate repeatability of the sensor’s performance.

**Figure 6:**
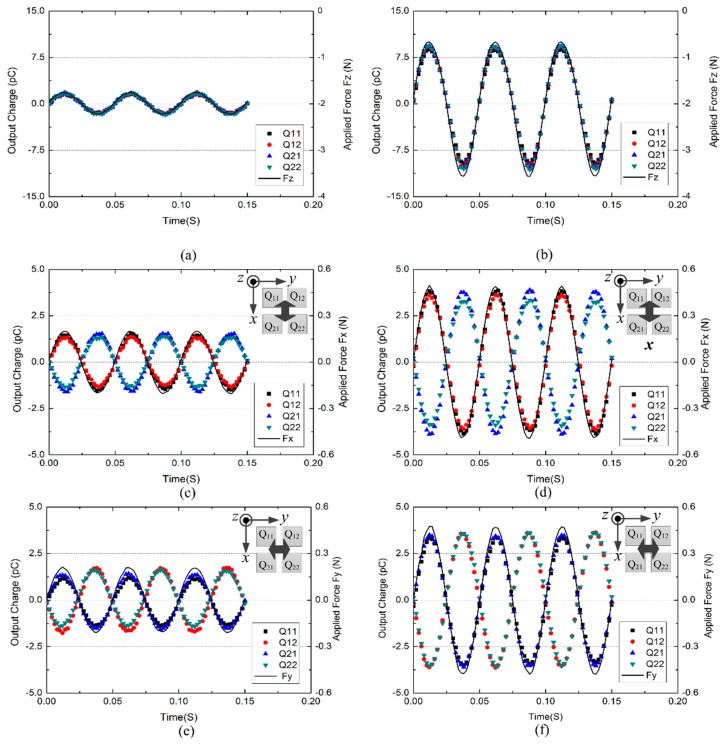
Measured charges of a tactile unit and applied forces in reference six-axis load cell when sinusoidal forces (*f* = 20 Hz) in normal and shear directions were applied respectively. (**a**) Normal force with amplitude F_zm_ = 0.24 N; (**b**) normal force with amplitude F_zm_ = 1.45 N; (**c**) shear force in x-axis with amplitude F_xm_ = 0.2 N; (**d**) shear force in x-axis with amplitude F_xm_ = 0.49 N; (**e**) shear force in y-axis with amplitude F_ym_ = 0.21 N; (**f**) shear force in y-axis with amplitude F_ym_ = 0.48 N.

Figure 6 presents measured tactile unit’s charge outputs and applied forces in reference six-axis load cell. Responses of the four piezoelectric capacitors were able to trace the applied forces well in all tests. From results of normal load tests showed in Figure 6a,b, we can observe a nearly uniform behavior of output charges from all four piezoelectric capacitors in a tactile unit. Comparing Figure 6b with Figure 6a, we can find that the amplitude of output charge increases with the amplitude of the input force. As for the results of shear load tests showed in Figure 6c–f, the polarity of the output charges $Q_{11}$ and $Q_{12}$ is opposite to that of $Q_{21}$ and $Q_{22}$ for the x-axis force, and the polarity of output charges $Q_{11}$ and $Q_{21}$ is opposite to that of $Q_{12}$ and $Q_{22},\;$exactly as what we described in Section 2.2.

Normal and shear components ($F_{x},\;F_{y}\text{ and }F_{z}$) of the applied three-axis force can be decoupled by combining the four output charges in different forms, as described in Section 2.2. Combinational output charges are defined as follows:

(4) $$\left\{ \begin{array}{l} Q_{x}=\frac{\left(Q_{11}-Q_{21})+(Q_{12}-Q_{22}\right)}{2}\\ Q_{y}=\frac{\left(Q_{11}-Q_{12})+(Q_{21}-Q_{22}\right)}{2}\\ Q_{z}=\frac{Q_{11}+Q_{12}+Q_{21}+Q_{22}}{4} \end{array}\right.$$

**Figure 7:**
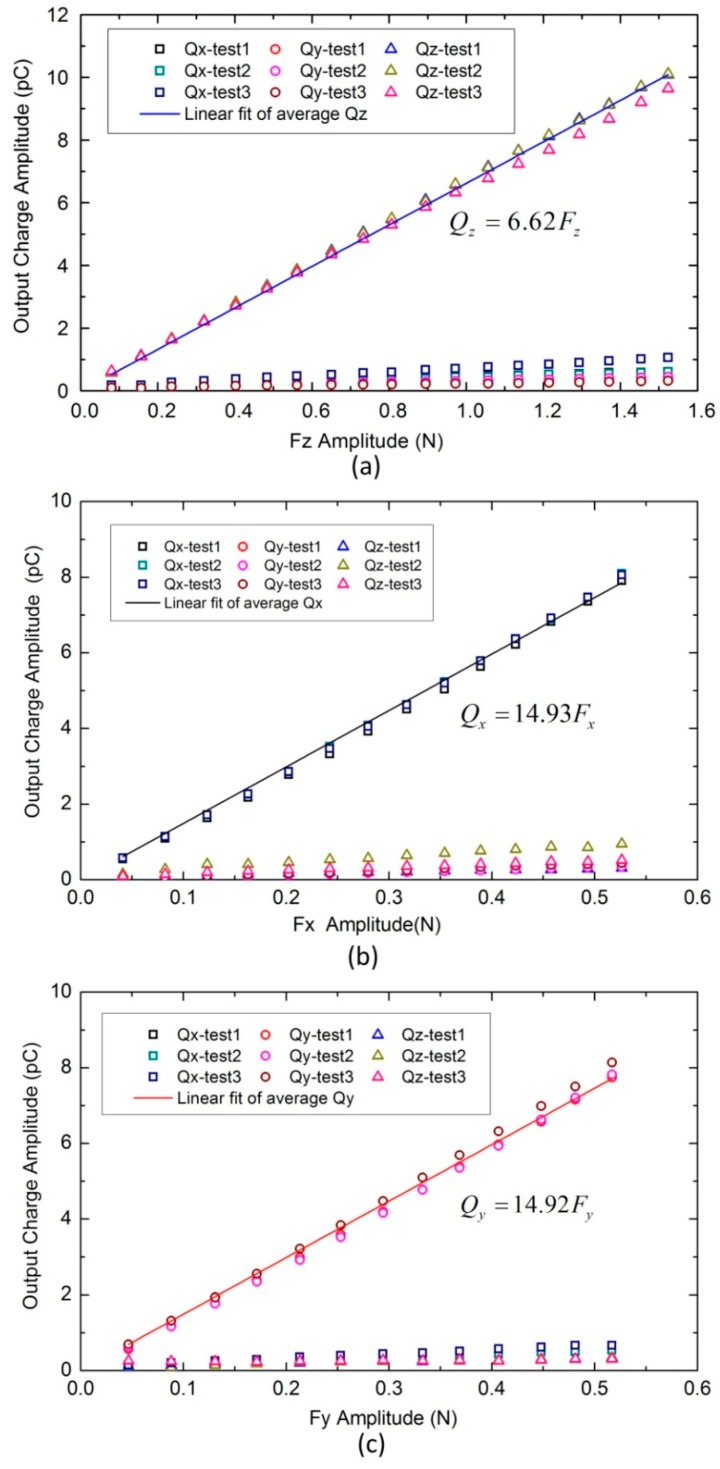
Average amplitudes (100 wave periods) of sinusoidal output charges *versus* sinusoidal input forces (20 Hz). In which symbols ‘□’, ‘○’, ‘△’ represent combinational output charges $Q_{x},\;Q_{y},\text{ and }Q_{z}$ , respectively. Each symbol has three colors indicating responses from three repeated tests. Solid lines represent linear fit of average response of the three repeat tests. (**a**) z-axis; (**b**) x-axis; (**c**) y-axis.

Using data in Figure 6, relationships between combinational output charges ($Q_{x},\;Q_{y},\text{ and }Q_{z}$) and applied forces were plotted in Figure 7. These plots relate average amplitudes (100 wave periods) of sinusoidal combinational output charges and corresponding average amplitudes (100 wave periods) of sinusoidal applied forces. Symbols ‘□’, ‘○’, ‘△’ represent combinational output charges $Q_{x},\;Q_{y},\text{ and }Q_{z}$, respectively. Each symbol has three colors indicating responses from three repeated tests. From Figure 7a we can see that the change in normal force mainly causes change in $Q_{z}$. From Figure 7b,c, we can see that the change in shear force mainly shows change in the corresponding combinational output charge (for instance, when shear force in the x-axis is increased, $Q_{x}$ changes with it). These mean cross-talk effects among the three axes are relatively small. Meanwhile, little deviations were observed from responses of three repeat tests, and the calculated repeatability in measuring x-, y-, and z-directional forces is 0.82%, 1.99%, and 1.35% F.S. (Full Scale), respectively. To evaluate the linearity, least squares linear curves were used to fit average responses of the three repeat tests. They all show good linearity, and the nonlinearity in measuring x-, y-, and z-directional forces is 2.45%, 2.37%, and 1.74% F.S., respectively. Slopes of fitted lines, which represent sensitivities of the tactile unit, have been extracted as 14.93, 14.92, and 6.62 pC/N for x-, y-, and z-axes, respectively. Using these values, three components of the applied force can be calculated as follows:

(5) $$\left\{ \begin{array}{l} F_{x}=\frac{1}{14.93}Q_{x}=0.067Q_{x}\\ F_{y}=\frac{1}{14.92}Q_{y}=0.067Q_{y}\\ F_{z}=\frac{1}{6.62}Q_{z}=0.1511Q_{z} \end{array}\right.$$

### 4.2. Frequency Effect Test

To evaluate frequency effect, the normal and shear load tests described in Section 3 have been repeated by altering the frequency in the range of interest of the present applications (f = 5 Hz, 20 Hz,100 Hz, 200 Hz and 400 Hz). This frequency range is suitable for several sensing applications. In the human hand, for example, the two types of fast–adapting mechanoreceptors FAI and FAII, which respond to dynamic events, such as contact, slippage, and texture encoding, are sensitive to dynamic skin deformation in the frequency ranges of ~5 to 50 Hz and ~40 to 400 Hz, respectively [1].

**Figure 8:**
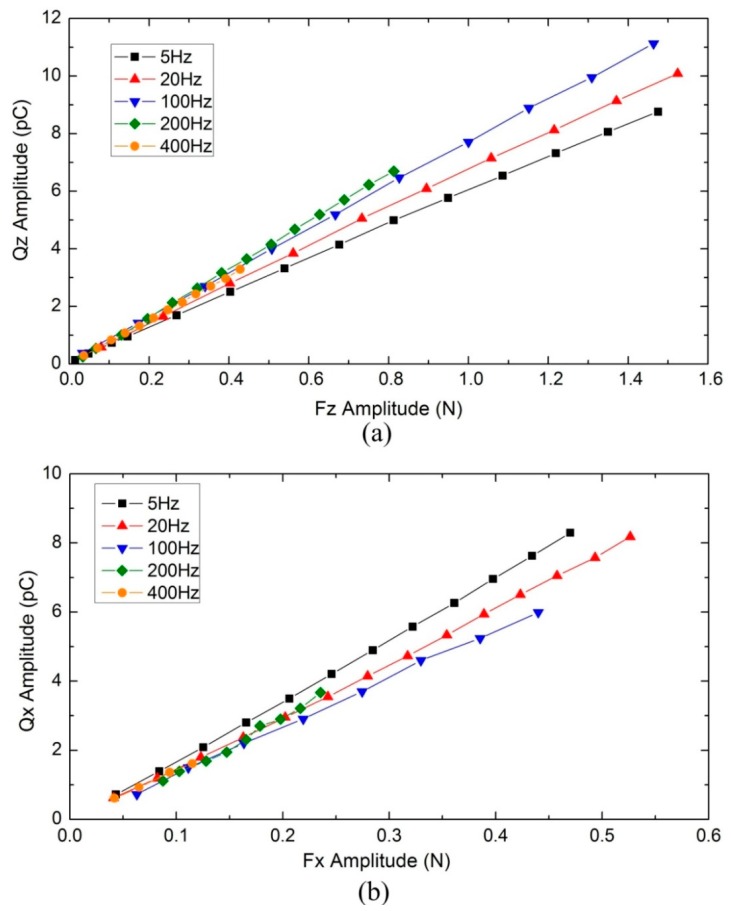
Frequency response of the tactile unit. (**a**) Average amplitude (100 wave periods) of sinusoidal output charge $Q_{z}$ *versus* sinusoidal input force $F_{z}$ at different frequencies; (**b**) average amplitude (100 wave periods) of sinusoidal output charge $Q_{x}$ *versus* sinusoidal input force $F_{x}$ at different frequencies.

Figure 8 shows average amplitude (100 wave periods) of sinusoidal combinational output charges *versus* corresponding average amplitude (100 wave periods) of sinusoidal applied forces at different frequencies. As it can be seen, a linear behavior is achieved over the whole explored range (5–400 Hz). The slope of charge–to–force curve, which represents sensitivity of the tactile unit, has small variation over the entire range of tested frequency in both normal and shear load tests. Overall, in normal direction the minimum sensitivity is 80% of the maximum sensitivity, and in the shear direction, the minimum sensitivity is 78% of the maximum sensitivity. According to the 3 db bandwidth requirement, our developed tactile unit shows acceptable frequency characteristic from 5 to 400 Hz in both normal and shear directional tests. Though the variation of sensitivity in the entire range of tested frequency is relatively small, an interesting variation tendency was observed in the frequency range from 5 to 100 Hz. It can be noticed that the sensitivity in the normal direction increased gradually when the frequency was changed from 5 to 100 Hz, and then remain constant. This may be attributed to the high-pass filtering effect of the charge amplifier. Contrarily, the sensitivity in shear (x-axis) direction decreased gradually when the frequency was changed from 5 to 100 Hz. This may be because the PDMS bump experiences greater deformation in the shear loading test than in the normal loading test, which induces a more significant damping effect.

### 4.3. Dynamic Three-Axis Force Measurement

**Figure 9:**
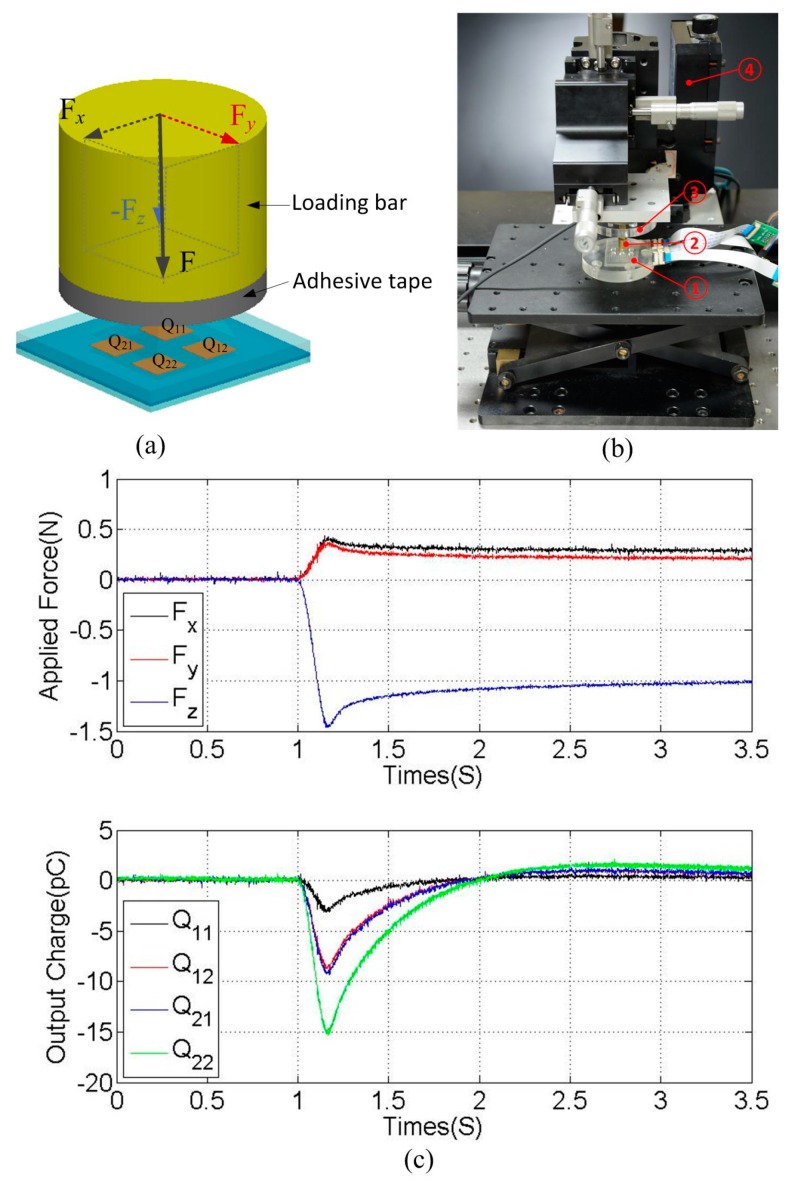
(**a**) Illustration of applying three-axis force on each unit of the sensor array; (**b**) photograph of the force loading system (① the fabricated tactile sensor array, ② the brass loading bar, ③ the six-axis load cell, ④ three-axial motion platform); and (**c**) response of a tactile unit to a three-axis force.

In the calibration tests, external force was applied on three axes separately to develop the data–fitted model in Equations (4) and (5). Actually, in a dexterous manipulation, force components in three axes are always applied simultaneously, forming a three-axis resultant force. To verify the developed data–fitted model in the reconstruction of the applied three-axis force, a dynamic three-axis force measurement has been conducted. The illustration and photograph of experimental setup is shown in Figure 9a,b. The brass loading bar is utilized to apply a dynamic three-axis force on each tactile unit. Adhesive tape is adhered to the bottom surface of the brass bar. When the bar contacts with the top surface of the bump, the tape sticks to the bump. Then, by automatically moving the bar in the x-, y- and z-axes simultaneously, a dynamic three-axis force F, which is composed of force components $F_{x},\;F_{y}\text{ and }F_{z}$, will be applied on the tactile unit. The brass bar is attached to the six-axis load cell which is mounted on an electric three-axis motion platform (M-VP-25XL-XYZ, Newport, RI, USA), as shown in Figure 9b. The motion platform can deliver highly reliable positioning performance at a maximum speed of 25 mm/s. Magnitudes of applied force components can be measured by the six–axis load cell, while output charges ($Q_{11}$, $Q_{12}$, $Q_{21}$ and $Q_{22}$) of the tactile unit are measured by the charge amplifier.

Different dynamic three-axis forces were applied on each tactile unit $U_{ij}$ (tactile unit located at the *i-th* row and *j-th* column, as shown in Figure 1a, *i* = 1, 2, 3, *j* = 1, 2). Figure 9c shows a typical response from one of the tests, in which a three-axis force composed of force components $F_{x}=0.4\;N，F_{y}=0.36\;N，\text{and }F_{z}=-1.45\;N$ was applied on tactile unit $U_{12}$. The variation of applied force measured by the load cell was shown in the upper half of Figure 9c, and responses from four piezoelectric capacitors were shown in the bottom half of Figure 9c. It can be seen that when the applied force changes, output charges $Q_{11},\;Q_{12},\;Q_{21}\text{ and }Q_{22}$ change correspondingly at the same time. By substituting $Q_{11},\;Q_{12},\;Q_{21}\text{ and }Q_{22}$ in Equations 4 and 5, three force components $F_{x},\;F_{y}\text{ and }F_{z}$ can be calculated.

Table 1 summarizes applied and calculated forces from all the tests. Percentage errors, calculated as $|(F_{calc}-F_{appl})/F_{appl}|\times 100\%$ ($F_{calc}$ is the calculated force component, $F_{appl}$ is the applied force component) are also presented. It can be seen that the calculated forces basically match the applied forces. However, several relatively large errors were generated as the applied force components are small. The maximal error as large as 37.5% was observed when an x-axis force component as small as 0.08 N was applied. Since Equations (4) and (5) are data-fitted models, errors always exist between the data points and the fitted curves. Meanwhile, errors brought by noise also always exist. So, when the applied force component is small, it is more likely to generate large percentage errors because the applied force component gets close to the error of the fitted-model and noise. Overall, the average error is 10.68% with a standard deviation of 6.84%. This error is acceptable in applications aiming to mimic human tactile sensing. It is reported that the just noticeable difference (JND) estimated from the sensation produced by passive compression of human finger skin ranges from 89.29% to 22.92% of applied basic weight when the basic weight changes from 20 to 200 g (0.2 to 2 N) [26].

### 4.4. Signal-to-Noise Ratio (SNR) Analysis

**Figure 10:**
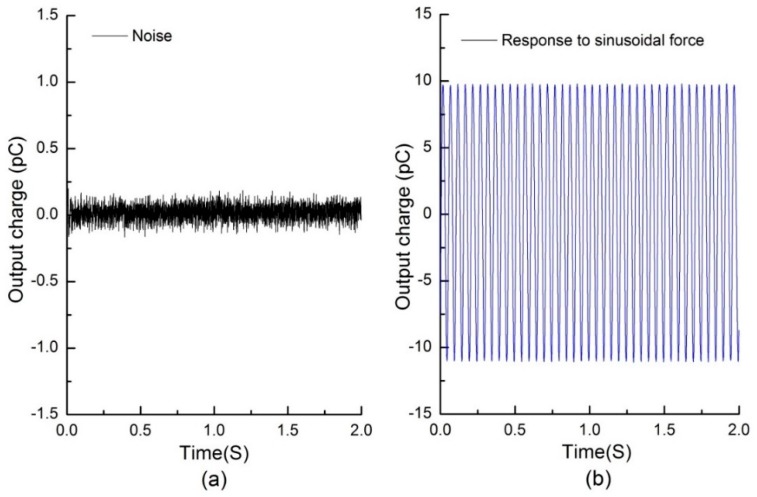
(**a**) The response of a piezoelectric capacitor in a tactile unit when no force was applied; and (**b**) the response of a piezoelectric capacitor when a 20 Hz sinusoidal force in z-axis direction with amplitude of about 5 N was applied on top surface of the tactile unit.

To evaluate the influence of the noise on measurement accuracy, signal-to-noise ratio analysis was carried out. Figure 10a shows the response of a piezoelectric capacitor in a tactile unit when no force was applied. Figure 10b shows response of the piezoelectric capacitor when a 20 Hz sinusoidal force in the z-axis direction was applied on the top surface of the tactile unit using the experimental setup and process described in the sensor unit calibration tests. The signal-to-noise ratio calculated by Equation (6) is 40.35 dB.

(6) $$SNR=10\log(\frac{P_{s}}{P_{n}})$$

where SNR is the signal-to-noise ratio of the sensor, $P_{s}$ is the power of the signal, and $P_{s}$ is the power of the noise.

## 5. Conclusions

In this paper, we have successfully demonstrated a new flexible dynamic three-axis tactile sensor array using PVDF film as the sensing material. The PVDF film in the array is horizontally arranged, which makes the fabrication process compatible with surface micromachining technology, enabling accurate control of sensor sizes and good spatial resolution.

A preliminary prototype sensor with 3 × 2 tactile units has been fabricated and tested. The results of calibration experiments demonstrate that the tactile unit has good linearity when sinusoidal stimulations with amplitudes in the range of 0–0.5 N, 0–0.5 N and 0–1.5 N were applied on x-, y- and z-axes separately. The calculated nonlinearities for x–, y– and z–axes are 2.45%, 2.37% and 1.74% F.S., respectively. The estimated sensitivities measured with the current setup are 14.93, 14.92 and 6.62 pC/N for x-, y- and z-axes, respectively. Furthermore, the sensor shows relatively low coupling effect, high repeatability, and acceptable frequency response from 5 to 400 Hz in both normal and shear load tests. By the results of calibration experiments, a date-fitted model has been established to calculate three components of the applied dynamic three-axis force. The calculated force components have a discrepancy of 10.68 ± 6.84% from the applied three-axis forces.

Obviously, structural dimensions of the sensor will affect the sensing performance of the developed sensor array. For example, a higher bump results in a larger torque when a shear force is applied on the top of the bump, which will increase the sensitivities for the x- and y-axes. Therefore, structural dimensions of the sensor will be optimized in our future work. Meanwhile, slippage and texture encoding in artificial hand applications will also be focused on. In addition, external noise is a source of measurement errors in our sensor, especially when the applied force is small. This problem will be paid attention to in future. One of the effective solutions is to add an electromagnetic shield on the surface of the sensor. 

## Figures and Tables

**Table 1:** Applied and calculated force components on the sensor array.

Tactile Unit	*F_x_*			*F_y_*			*F_z_*		
	Appl.	Calc.	|Err.|	Appl.	Calc.	|Err.|	Appl.	Calc.	|Err.|
	(N)	(N)	(%)	(N)	(N)	(%)	(N)	(N)	(%)
U_11_	0.16	0.15	6.25	0.22	0.19	13.64	−0.595	−0.55	7.56
U_11_	0.34	0.29	14.71	0.33	0.30	9.09	−0.95	−0.885	6.84
U_12_	0.08	0.11	37.50	0.32	0.30	6.25	−0.695	−0.655	5.76
U_12_	0.40	0.42	5.00	0.36	0.39	8.33	−1.45	−1.35	6.90
U_21_	0.23	0.19	17.39	0.08	0.05	37.50	−0.545	−0.495	9.17
U_21_	0.12	0.14	16.67	0.45	0.49	8.89	−1.02	−0.92	9.80
U_22_	0.43	0.46	6.98	0.41	0.43	4.88	−1.32	−1.2	9.09
U_22_	0.34	0.30	11.76	0.12	0.14	16.67	−0.745	−0.63	15.44
U_31_	0.15	0.16	6.67	0.19	0.17	10.53	−0.475	−0.38	20.00
U_31_	0.21	0.22	4.76	0.44	0.39	11.36	−0.99	−1.05	6.06
U_32_	0.44	0.45	2.27	0.16	0.15	6.25	−0.96	−0.89	7.29
U_32_	0.20	0.19	5.00	0.29	0.30	3.45	−0.73	−0.665	8.90
